# Diet quality and Gleason grade progression among localised prostate cancer patients on active surveillance

**DOI:** 10.1038/s41416-019-0380-2

**Published:** 2019-01-25

**Authors:** Justin R. Gregg, Jiali Zheng, David S. Lopez, Chad Reichard, Gladys Browman, Brian Chapin, Jeri Kim, John Davis, Carrie R. Daniel

**Affiliations:** 1 Department of Urology, The University of Texas MD Anderson Cancer Center, Houston, TX 77030 USA; 2 Department of Epidemiology, The University of Texas MD Anderson Cancer Center, Houston, TX 77030 USA; 3 Department of Preventive Medicine and Community Health, The University of Texas Medical Branch, Galveston, TX 77555 USA

**Keywords:** Prostate cancer, Cancer epidemiology, Epidemiology

## Abstract

**Background:**

High diet quality may support a metabolic and anti-inflammatory state less conducive to tumour progression. We prospectively investigated diet quality in relation to Gleason grade progression among localised prostate cancer patients on active surveillance, a clinical management strategy of disease monitoring and delayed intervention.

**Methods:**

Men with newly diagnosed Gleason score 6 or 7 prostate cancer enroled on a biennial monitoring regimen. Patients completed a food frequency questionnaire (FFQ) at baseline (*n* = 411) and first 6-month follow-up (*n* = 263). Cox proportional hazards models were fitted to evaluate multivariable-adjusted associations of diet quality [defined via the Healthy Eating Index (HEI)-2015] with Gleason grade progression.

**Results:**

After a median follow-up of 36 months, 76 men progressed. Following adjustment for clinicopathologic factors, we observed a suggestive inverse association between baseline diet quality and Gleason grade progression [hazard ratio (HR) and 95% confidence interval (CI) for the highest vs. the lowest HEI-2015 tertile: 0.59 (0.32–1.08); *P*_trend_ = 0.06]. We observed no associations with diet quality at 6-month follow-up, nor change in diet quality from baseline.

**Conclusions:**

In localised prostate cancer patients on surveillance, higher diet quality or conformance with United States dietary guidelines at enrolment may lower risk of Gleason grade progression, though additional confirmatory research is needed.

## Introduction

Prostate cancer is the most commonly diagnosed and second leading cause of cancer death among men in the United States^1^ The majority of men diagnosed with prostate cancer (91%) are found to have a local or regional stage disease, for which the 5-year survival rate approaches 100%.^1^ However, there is substantial concern with regards to potentially life-altering overtreatment among men diagnosed with early-stage prostate cancer; and, use of active surveillance (AS) as an initial treatment option for these lower-risk patients is increasing.^2–4^ An estimated 50% of prostate cancer patients may be eligible for this initial therapy to avoid or delay treatment until there is evidence of disease progression.^3^ While multiple clinical factors, such as serum prostate-specific antigen (PSA), biopsy results (pathologic Gleason score, tumour volume), and age at diagnosis, have been linked to disease progression on surveillance, they are not inherently modifiable.^5,6^ Many single dietary factors or nutrients, such as calcium and Vitamin D, have been assessed in the setting of prostate cancer risk, including advanced or aggressive disease,^7–9^ but there are no clear, evidence-based dietary recommendations to inform men diagnosed with localised prostate cancer how to lower their risk of progression during AS.

The 2018 Third Expert Report of the World Cancer Research Fund/American Institute for Cancer Research emphasises that different patterns of diet create a metabolic and inflammatory state that is more or less conducive to tumour progression, while specific foods or nutrients are less likely to be important singular factors in promoting or inhibiting cancer.^10^ The collective evidence shows that individuals with the highest adherence to dietary recommendations (or diet quality) experience the greatest reductions in cancer morbidity and mortality.^10^ Several large prospective studies have shown that high diet quality is associated with lower risk of developing prostate cancer.^11–13^ Given the paucity of data regarding dietary habits of newly diagnosed, early-stage prostate cancer patients and the potential impact of diet quality on disease progression in this population,^14^ we assessed diet in men enroled on an AS protocol and followed for Gleason grade progression over a biennial monitoring regimen. We derived the Healthy Eating Index (HEI)-2015, reflecting adherence to established dietary guidelines for Americans across 13 dietary components, and prospectively examined baseline diet quality in relation to progression-free survival (PFS). We additionally explored the effect of diet quality at 6-month clinical follow-up and respective change from baseline with PFS.

## Methods

### Study design and population

The AS protocol, designed to safely monitor men for disease progression and evaluate clinical risk stratification, was conducted by a multidisciplinary team of urologic surgeons, radiation oncologists and medical oncologists at MD Anderson Cancer Center (MDACC). This observational study of patients on AS is registered on clinical.trials.gov (NCT00490763), was approved by the Institutional Review Board and required all patients to provide informed consent. Protocol criteria including surveillance frequency and details regarding disease diagnostic upstaging are available elsewhere.^15,16^ A total of 560 MDACC patients who were diagnosed with Gleason score 6 or 7 localised prostate cancer were enroled into this prospective clinical cohort between February 2006 and February 2012. Among these, 501 provided a baseline food frequency questionnaire (FFQ). Following exclusion of patients with extreme total energy intake (defined as beyond twice the interquartile range of Box–Cox transformed intake, *n* = 53), 411 of the remaining patients stayed on AS for at least 6 consecutive months and were included in the final analysis. A subset of these patients (*n* = 263) completed another FFQ at the first 6-month clinical follow-up.

### Assessment of diet quality and patient characteristics

Usual dietary intake at baseline and follow-up was assessed using a 170-item modified Block FFQ, as described previously.^16–18^ FFQs were reviewed and coded by trained registered dietitians for completeness and acceptability. Total energy and nutrient intake were calculated by linking FFQ responses (frequency and portion size) to the US Department of Agriculture (USDA) Food and Nutrient Database for Dietary Studies.^19^ Food groups or pyramid equivalents were derived as described previously.^18^ Diet quality was defined using the latest version of the HEI-2015 that aligns with the USDA’s 2015–2020 dietary guidelines for Americans and encompasses 13 components, including 9 adequacy components for which higher intake receives a higher score and 3 moderation components which are reverse scored (i.e. higher intake receives lower score^20^). The nine adequacy components are total fruits, whole fruits, total vegetables, greens and beans, whole grains, dairy, total protein foods, seafood and plant proteins and unsaturated to saturated fatty acids ratio. Three moderation components are refined grains, sodium, added sugars and saturated fats. Briefly, each of the HEI-2015 components (Supplemental Table 1) was scored on an energy density basis per 1000 kcal or as a percentage of energy (for added sugars and saturated fats), with the exception of the fatty acids ratio. For each component, individuals’ intake was scored at a range from the minimum score (0) to the maximum (5 or 10) for a total overall score of up to 100 points.^20^ A higher HEI-2015 score indicates better adherence to the 2015–2020 dietary guidelines for Americans and thus higher overall diet quality, while a lower score indicates lower diet quality.

Baseline serum PSA, pathologic Gleason score, and summary tumour length (defined as the sum of diagnostic and confirmatory biopsy tumour length) were assessed at study enrolment.^15^ Measured height and weight, smoking status, and health history were drawn from the medical record. Body mass index (BMI) was calculated as weight (kg)/height (m)^2^ and categorised based on the World Health Organisation criteria.

### Assessment of outcomes

Patients underwent a confirmatory biopsy at study entry, with rare exception, and were evaluated every 6 months through clinical examination (digital rectal exam) and laboratory studies (serum PSA, testosterone). Prostate biopsies were repeated every 1–2 years; if a biopsy was negative, then the following year’s biopsy was omitted. All biopsies were performed using the trans-rectal ultrasound-guided technique with an 11-core multisite-directed biopsy scheme.^21^ Patients who had disease reclassification, defined as increase in tumour volume (core number or percentage) or Gleason increase, were recommended to undergo treatment, though patients who wished to remain on surveillance were allowed to do so if approved by their treating physician. The primary outcome of interest was grade progression, defined as any increase in Gleason score following confirmatory biopsy. PSA kinetic changes, alone, were not used to reclassify patients. Patients were followed until grade progression, treatment, loss to follow-up, elective study removal, death or 31 December 2016 (the censor date of the study), whichever came first.

### Statistical analysis

Diet quality (HEI-2015) score at baseline and at 6-month clinical follow-up was categorised into tertiles (high, medium, low) based on the population distribution. Differences across tertiles for continuous and categorical variables were tested using analysis of variance and *χ*^2^ tests, respectively. We evaluated the association between diet quality and PFS by using Cox proportional hazard (PH) regression models with person-years as the underlying time metric. Hazard ratios (HR), 95% confidence intervals (CI) and *P* values for linear trend (using the median value within tertiles) are reported across high, medium, and low diet quality, with the lowest HEI-2015 tertile representing the referent group. We confirmed the PH assumption was met through assessment of interaction terms for the exposures with follow-up time.

We examined two models in the analyses: (1) age- and energy-adjusted model (base model)^22^ and (2) a model additionally adjusted for baseline clinicopathologic factors including PSA and summary tumour length (“base + clinical characteristics model”). We additionally evaluated other clinical, lifestyle, and demographic factors potentially related to diet quality and grade progression, including tumour stage, smoking status, race, BMI, hypertension, diabetes, alcohol consumption and statin use. In the 6-month follow-up and proportional change score (percentage change of HEI-2015 score from baseline to 6-month follow-up) analysis, we additionally examined baseline diet quality, as a covariate. However, none of these factors appreciably modified the crude hazard ratio or the final models, and thus were not included. To evaluate whether any single component of diet quality was driving the overall association, each of the 13 HEI-2015 component scores were assessed individually, and mutually adjusted, in the base + clinical characteristics model. We additionally assessed whether the association between diet quality and grade progression varied by baseline clinicopathologic, lifestyle and demographic factors, including circulating testosterone level, age group, race, smoking status, BMI, alcohol consumption and chronic conditions/medication use. Statistical tests for interaction evaluated the significance of categorical cross-product terms in the multivariable-adjusted models. All statistical tests were two-sided and were considered statistically significant at *P* < 0.05. Statistical analyses were conducted using STATA version 13.1 (StataCorp, College Station, TX, USA) and SAS (version 9.4, Cary, NC, USA).

## Results

Baseline characteristics by high, medium and low diet quality (tertiles of HEI-2015) score are displayed in Table 1. The mean HEI-2015 score at study entry was 67.4 (SD = 10.5). Compared to the patients with the lowest baseline diet quality, patients with the highest diet quality were older, had a lower BMI, and reported lower total energy and alcohol intake. While majority of men at baseline were Gleason 6 and within the National Comprehensive Cancer Network criteria for “very-low-risk” to “low-risk,” Gleason 7 patients tended to report higher diet quality. In the subset of patients (64.0%; 263/411) that additionally completed an FFQ at 6-month clinical follow-up, the mean follow-up HEI-2015 score was 69.8 (SD = 9.3).

**Table 1:** Means and proportions for selected baseline characteristics of localised prostate cancer patients on active surveillance by baseline diet quality score^a^ (*n* = 411)

Characteristics^b^	Low diet quality (34.8–63.3)	Med diet quality (63.3–72.7)	High diet quality (72.9–95.1)	*P* value^c^
*N*	137	137	137	
Demographics				
Age (years)	62.5 (7.8)	65.0 (8.6)	65.7 (8.4)	<0.01
Race, *N* (%)				0.30
White	110 (80.3)	116 (84.7)	115 (83.9)	
Black	16 (11.7)	10 (7.3)	7 (5.1)	
Other/unknown	11 (8.0)	11 (8.0)	15 (10.9)	
Clinical features				
Baseline Gleason score, *N* (%)				0.09
Gleason 6	125 (91.2)	121 (88.3)	113 (82.5)	
Gleason 7	12 (8.8)	16 (11.7)	24 (17.5)	
PSA (ng/mL)	4.3 (2.3)	4.1 (2.7)	4.0 (2.7)	0.67
Summation tumour length (mm)^d^	3.1 (4.0)	3.8 (5.4)	3.6 (5.5)	0.58
Baseline core positivity^e^, *N* (%)				0.22
Single	104 (75.9)	92 (67.2)	102 (74.5)	
Multiple	33 (24.1)	45 (32.8)	35 (25.5)	
Lifestyle and health history				
Clinical T stage, *N* (%)				0.78
cT1	123 (89.8)	121 (88.3)	117 (85.4)	
cT2a	12 (8.8)	15 (10.9)	18 (13.1)	
cT2b or cT2c	2 (1.5)	1 (0.8)	2 (1.5)	
BMI (kg/m^2^)	29.3 (4.6)	28.4 (4.1)	27.9 (4.0)	0.02
Total energy intake (kcal/day)	2580 (1179)	2381 (935)	2132 (766)	<0.01
Alcohol intake level (drinks/week)				0.06
None	42 (30.7)	33 (24.1)	37 (27.0)	
0.1–4.1	59 (43.1)	47 (34.3)	44 (32.1)	
4.1–57.0	36 (26.3)	57 (41.6)	56 (40.1)	
Smoking status				0.28
Ever	84 (61.3)	71 (51.8)	78 (56.9)	
Never	53 (38.7)	66 (48.2)	59 (43.1)	
Statin use				0.68
Yes	59 (43.1)	57 (41.6)	64 (46.7)	
No	78 (56.9)	80 (58.4)	73 (53.3)	
Hypertension				0.93
Yes	70 (51.1)	67 (48.9)	68 (49.6)	
No	67 (48.9)	70 (51.1)	69 (50.4)	
Diabetes mellitus				0.59
Yes	20 (14.6)	17 (12.4)	23 (16.8)	
No	117 (85.4)	120 (87.6)	114 (83.2)	
Testosterone (ng/dL)				0.27
<350	59 (43.1)	54 (39.4)	46 (33.6)	
≥350	78 (56.9)	83 (60.6)	91 (66.4)	

*BMI* body mass index, *ANOVA* analysis of variance, *PSA* prostate-specific antigen, *HEI* Healthy Eating Index

^a^Diet quality is defined by the HEI-2015 score categorised into tertiles

^b^Presented as mean and standard deviation, unless otherwise specified *N* (%). Sum of percentages may not add up to 100% due to rounding

^c^Statistical analyses were performed by ANOVA test for continuous variables and by *χ*^2^ test for categorical variables

^d^Baseline tumour length (sum of tumour length from diagnostic and confirmatory biopsies)

^e^Number of positive cores detected on the diagnostic biopsy

Over a median follow-up of 36 months (range 6–126 months), 18.5% of patients (76/411) experienced grade progression, and 12 patients died of other causes without documented progression. Although the mean baseline HEI-2015 score was significantly higher in patients who did vs. did not comply with the follow-up dietary assessment (mean = 68.5, SD = 9.8 vs. mean = 65.5, SD = 11.5; *P*-diff = 0.005), the progression rate did not differ between these groups (19.4% vs. 16.9%, *P*-diff = 0.60).

Multivariable-adjusted models evaluating the association between baseline diet quality and PFS are shown in Table 2. Following adjustment for age, total energy intake and clinical characteristics, we observed a suggestive inverse association between high baseline diet quality and PFS (HR_T3 vs. T1_ = 0.59, 95% CI = 0.32–1.08, *P*_trend_ = 0.06). Neither diet quality at 6-month follow-up (HR_T3 vs. T1_ = 1.05, 95% CI = 0.54–2.04, *P*_trend_ = 0.67) nor proportional change in diet quality from baseline to 6-month follow-up (HR_improve vs. decline_ = 1.10, 95% CI = 0.59–2.05, *P* = 0.76) was associated with PFS. We additionally examined if any of the 13 individual HEI-2015 components were driving the overall association observed, and although many were in a consistent and expected direction, none were significantly associated with PFS on their own (data not shown).

**Table 2:** HRs and 95% CIs for the association between diet quality^a^ and disease progression (Gleason score upgrading) in localised prostate cancer patients on active surveillance

	Range	*N*	Events	Base Model^b^			Base + Clinical Characteristics Model^c^		
				HR	95% CI	*P* value	HR	95% CI	*P* value
Baseline diet quality									
Low	34.81–63.30	137	29	1.00	Ref.	Ref.	1.00	Ref.	Ref.
Med	63.32–72.73	137	28	0.90	0.53–1.53	0.70	0.90	0.52–1.53	0.69
High	72.87–95.14	137	19	0.62	0.34–1.12	0.11	0.59	0.32–1.08	0.09
*P*_trend_^d^						0.11			0.06
6-Month clinical follow-up diet quality									
Low	38.97–65.08	88	19	1.00	Ref.	Ref.	1.00	Ref.	Ref.
Med	65.17–74.45	88	13	0.66	0.32–1.33	0.24	0.77	0.37–1.60	0.49
High	74.66–93.84	87	19	0.99	0.52–1.89	0.98	1.05	0.54–2.04	0.90
*P*_trend_^d^						0.94			0.67
Proportional change in diet quality from baseline to 6-month follow-up									
Decline	−0.15% to −25.4%	116	21	1.00	Ref.	Ref.	1.00	Ref.	Ref.
Improve	0.02–29.6%	147	30	1.10	0.59–2.05	0.76	1.18	0.62–2.25	0.62

*BMI* body mass index, *HR* hazard ratio, *CI* confidence interval, *ANOVA* analysis of variance, *PSA* prostate-specific antigen, *HEI* Healthy Eating Index

^a^Baseline diet quality was defined by baseline HEI-2015 assessed in all 411 patients; 6-month clinical follow-up diet quality was defined by post-diagnostic HEI-2015 in a subset of 263 patients; and proportional change in diet quality from baseline to 6-month follow-up was calculated as the HEI-2015 score change from baseline to 6 months (baseline–6 months)

^b^Base Model adjusted for age and total energy intake

^c^Base + Clinical Characteristics model additionally includes PSA and composite tumour length

^d^*P*_trend_ was calculated by using the median of each tertile as a continuous variable in the Cox proportional hazard model

**Fig. 1:**
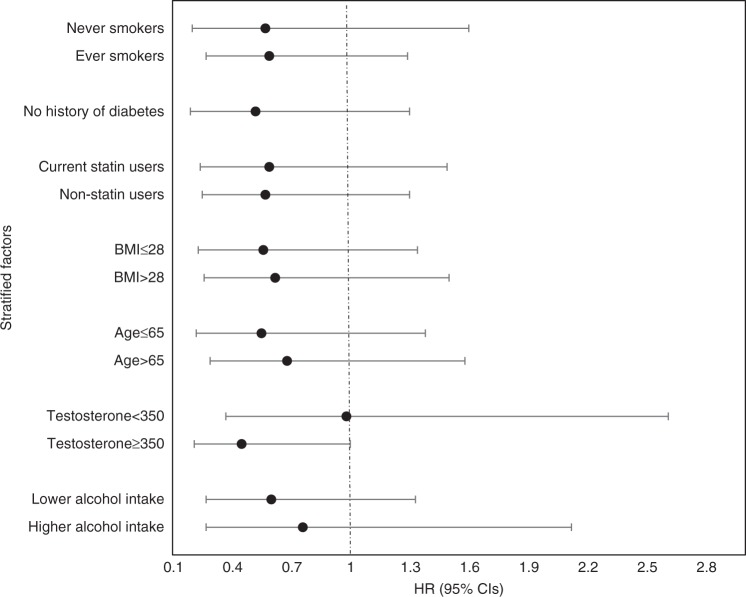
Association between baseline diet quality and disease progression according to selected characteristics. Risk of progression comparing the highest vs. the lowest (referent) tertile of the baseline Healthy Eating Index (HEI)-2015. All *P*-interaction >0.05

Figure 1 shows the multivariable-adjusted associations between baseline diet quality and PFS according to a priori selected potential effect modifiers. No statistically significant interactions were observed and the modest inverse relationship between diet quality and PFS appeared fairly consistent. Notably the protective effect of diet quality appeared more pronounced among men with higher circulating testosterone levels [350 ng/dL or above; HR and 95% CI: 0.45 (0.21–1.00)]. No association was observed among men with lower testosterone levels [<350 ng/dL; HR and 95% CI: 0.98 (0.37–2.61)]. We additionally performed a sensitivity analysis among white men only (given limited numbers of men of other races) and observed similar, but non-significant findings [HR_T3 vs. T1_ = 0.59, 95% CI = 0.47–1.80, *P*_trend_ = 0.82)].

## Discussion

In a prospective cohort of men with localised prostate cancer enroled on an AS protocol, baseline diet quality, as measured by the HEI-2015 score, appeared to be associated with lower risk of grade progression. This would suggest that consistently following a healthful dietary pattern characterised by a variety of fibre-rich plant foods (e.g. vegetables, whole fruits, legumes, whole grains) and healthy balance of unsaturated fats, while minimising saturated fats, added sugars and refined grains, may be beneficial for men diagnosed with early-stage prostate cancer. Although none of our findings reached statistical significance, the direction of this inverse relationship appeared to be consistent across a range of lifestyle and clinical factors. Diet quality measured at 6-month clinical follow-up in a subset of patients, and proportional change in diet quality between baseline and 6-month follow-up was not associated with PFS, suggesting that usual, healthy dietary habits as opposed to short-term changes may be more promising in this setting.

Dietary patterns that score well across a range of parameters and international recommendations (e.g. Healthy Eating Index and Mediterranean Diet Score) are associated with significantly lower risk of developing and dying from cancer.^23,24^ Several epidemiological studies have investigated the association between index-based dietary patterns and risk of prostate cancer.^11–13^ In the large, US prospective National Institutes of Health-American Association of Retired Persons study, higher diet quality, as defined by HEI-2005 or the Alternate HEI-2010, was significantly associated with lower prostate cancer risk.^11^ Similar findings were reported in the Health Professionals Follow-up study.^12^ Our study offers some of the first evidence that in addition to lowering the risk of developing prostate cancer, higher diet quality or adherence to dietary recommendations may also lower risk of grade progression in localised prostate cancer patients on AS. Interestingly, none of the individual components of the HEI were significantly associated with PFS, further supporting that when considering a balance of healthy and unhealthy dietary behaviours, the impact of the sum may be greater than its parts. Prior prospective investigations considering multiple dietary components or data-driven dietary patterns in men with prostate cancer are consistent with our findings. In over 4500 prostate cancer cases enroled in the Health Professionals Follow-up Study, Richman et al.^25^ modelled dietary changes and reported that substitution of 10% of energy intake from carbohydrate with vegetable fat could lower the risk of lethal prostate cancer, defined as distant metastases or prostate cancer-specific death by 29% (HR = 0.71, 95% CI = 0.51–0.98, *P* = 0.04).^25^ In the same cohort, a Western dietary pattern (characterised by higher intake of processed and red meats, high-fat dairy and refined grains), as compared to a Prudent dietary pattern (characterised by high intake of vegetables, fruits, fish, legumes, and whole grains), was associated with increased prostate cancer-specific and overall mortality.^14^

Our null findings for diet quality at 6-month clinical follow-up and short-term proportional change score in a small subset of patients should not discourage men from taking steps to improve their overall dietary habits following their prostate cancer diagnosis. Our findings for baseline diet quality and grade progression in men on AS are generally consistent with a small randomised trial evaluating a low-fat, plant-based diet in conjunction with increased physical activity, which reported decreased need for treatment intervention at 2 years.^26^ A recently reported phase III randomised trial (The Men’s Eating and Living or MEAL study) evaluating a validated phone-based counselling programme to increase vegetable consumption in men on AS offers further evidence that overall diet quality, rather than individual components, may be relevant to men on AS.^27^ In the study, 237/478 men randomised to the treatment arm successfully increased vegetable intake; however, there was no difference in prostate cancer progression-free survival (HR 0.96, 95% CI 0.75–1.24, *P* = 0.76 for the treatment arm compared to the control).^28^ Taken together with our findings, short-term dietary modification following diagnosis and/or a focus on singular dietary factors may not be the most effective strategy in this setting.

A number of mechanisms support the potential protective effect of high diet quality in localised prostate cancer patients commencing AS. The antioxidant and anti-inflammatory properties of the multiple dietary components that constitute this score, including a variety of fibre-rich plants foods, low added sugars and healthy balance of unsaturated fats, may collectively support a systemic and tumour environment that inhibits progression.^29–33^ These mechanisms are further supported by findings from several dietary intervention trials with blood-based marker outcomes among men with prostate cancer, including PSA level, plasma cytokines, sex hormones and insulin-like growth factors.^34–38^ Although the HEI-2015 is designed to represent diet quality independent of diet quantity (total energy intake), its potential protective effect may also be realised through energy balance and obesity, an established risk factor for prostate cancer progression.^39^

Strengths of our study reside in the use of a prospective clinical AS protocol that includes an assessment of overall diet quality. The pre-specified surveillance protocol yielded robust data regarding features of the prostate cancer diagnosis and clinical outcome assessment, and represents the first investigation, to our knowledge, to examine adherence to current dietary guidelines (via the recently released HEI-2015) in men on AS. Despite the unique nature of the study, we are limited by sample size and length of follow-up, particularly when evaluating diet at 6-month clinical follow-up, a potentially critical window of behaviour change in newly diagnosed prostate cancer patients on AS. Although several important confounders were considered in the analyses, residual or unmeasured confounding, particularly by physical activity, is possible. Measurement error and recall bias in self-reported dietary data is another unavoidable limitation.

In summary, higher baseline diet quality or stricter adherence to US dietary guidelines may lower risk of grade progression in localised prostate cancer patients on AS. Our suggestive findings warrant follow-up in larger studies. Men on surveillance generally have an excellent cancer-related prognosis, but are also susceptible to other chronic diseases, including cardiovascular disease and other cancers, underscoring the importance of addressing risk factors, such as overall diet, that may affect multiple health outcomes.^40,41^ Thus, men should continue to be encouraged to adhere to healthy lifestyles and follow dietary guidelines both before and after localised prostate cancer diagnosis. With further validation, baseline diet quality may serve as an early prognostic marker to help guide surveillance frequency and patient referral for dietetic counselling in men on AS. However, in the short term, diet quality/adherence to existing dietary recommendations presents a readily translatable, broadly applicable and safe message to disseminate to patients.

## Supplementary information

Supplemental Table 1

## Data Availability

The datasets generated during and/or analysed during the current study used to support the findings of this study are not publicly available as this dataset is a resource of the University of Texas MD Anderson Cancer Centre; however, information is available from the corresponding author on reasonable request.
